# Clinical Outcomes of Radial Shortening Osteotomy and Vascularized Bone Graft in Kienböck's Disease

**DOI:** 10.1155/2014/956369

**Published:** 2014-11-09

**Authors:** Mohammad Dehghani, Mohammad Ali Moshgelani, Mohammad Hadi Nouraei, Shaghayegh Dehghani, Maryam Gholshahi

**Affiliations:** ^1^Department of Orthopedics, School of Medicine, Isfahan University of Medical Sciences, P.O. Box 81465-1148, Isfahan, Iran; ^2^Department of Radiology, School of Medicine, Isfahan University of Medical Sciences, P.O. Box 81745-319, Isfahan, Iran

## Abstract

The aim of this study was to compare two surgery methods including radial shortening and radial shortening combined with vascularized bone graft for treatment of stage II or IIIa of Kienböck's disease. It is a randomized, controlled clinical trial, which was carried out in 2011–2013. Twenty-four patients were assigned equally to radial shortening group (A) or radial shortening combined with vascularized bone graft group (B). The outcome was assessed by Mayo Wrist score before and 9 months after surgery. The mean Mayo Wrist score (SD) was 27.1 (15.4) and 32.5 (18.3) before surgery and 74.6 (5.4) and 85.8 (5.1) after surgery for groups A and B, respectively. The mean score increased in both groups, and it was higher in group B significantly. Radial shortening combined with vascularized bone graft is a valuable method which can be more effective than radial shortening alone, in early stages of Kienböck's disease. This trial is registered with IRCT201404127841N5.

## 1. Introduction 

Kienböck's disease is an unusual problem that causes osteonecrosis and collapse of the lunate bone, which leads to chronic dysfunction and pain. It is usually seen in adults between 20 and 40 years of age and is typically unilateral [1, 2]. Despite its long lasting recognition, the etiology and natural history of this disease remain unknown at present and multifactorial pathogenesis which is influenced by mechanical, anatomic, vascular, traumatic, and systemic factors has been proposed. Kienböck's disease displays a progressive nature and destructs the wrist joint, so prompt diagnosis and appropriate treatment are obligatory [2–4].

The treatment of this disease is a challenging problem. The goals of treatment include maintaining normal wrist kinematics, conservation of wrist function, and, when and if possible, revascularization of the necrotic bone. Several methods have been suggested in the literature for treatment of Kienböck's disease, but, based on previous data, no active treatment is superior in the treatment of this disease and there are neither homogeneous studies nor large series that have described which treatment is best [5–8]. Treatment modalities range from conservative methods such as immobilization to operative ones such as intercarpal fusions, excision, partial capitate shortening, implant arthroplasty, ulnar lengthening, radial shortening, and vascularized bone grafting [9–13].

When either of the conservative methods is unsuccessful or there is a more advanced stage, surgical procedure is recommended. In Lichtman stage II of disease, sclerosis of the lunate and/or early collapse of the lunate radial border may appear. In stage IIIA, there is more severe collapse. In stages II and IIIA, the remainder of the carpus is still uninvolved, so treatment involves surgeries for revascularization of the lunate either directly (with vascularized bone grafting) or indirectly (by unloading the lunate) [3, 14].

Distal radial shortening is one of the most commonly used procedures and recent reports of long-term outcomes of radial shortening osteotomy for earlier stages have shown that this method is a reliable treatment option for relieving pain and improving function [1, 7, 11, 14]. Since the most apparent predisposing factor of disease is the unsteady blood supply to the lunate, newer modalities such as vascularized bone grafts, which improve direct lunate revascularization, may arrest the progression of collapse. However, current data are not enough to conclude with certainty whether this type of surgeries shows more improvement over the traditional treatment choices [6, 14].

Some studies represented that there is no substantial clinical difference between the radial shortening and vascularized bone graft in long-term outcome [15]; but some suggested that decompression surgery along with revascularization may provide better results than either alone [16, 17].

In this study, our aim was to evaluate the outcome of surgery in patients with stage II or IIIa who were treated via radial shortening with or without vascular bone graft.

## 2. Materials and Methods

### 2.1. Study Design and Participants

This study used a randomized, controlled clinical trial design, which was carried out in 2011–2013 and posted on Iranian Registry of Clinical Trials (http://www.irct.ir/) with identifier number IRCT201404127841N5. The study followed the Declaration of Helsinki on Biomedical Research Involving Human Subjects and was approved by the Ethics Committee of the Isfahan University of Medical Sciences. All participants provided written informed consent.

Subjects were chosen from patients who were referred to the Kashani Hospital (Isfahan, Iran). All subjects met the following inclusion criteria: (1) aged 18–65 years, (2) diagnosed with stage II or IIIa of Kienböck's disease according to the Lichtman criteria [18], (3) diagnosed with ulnar minus variance, and (4) providing written informed consent. Subjects also met none of the following exclusion criteria: (1) any contraindication for receiving general anesthesia and (2) any serious medical condition that may interfere with safe study participation.

A total of 38 individuals who were clinically suspected to have Kienböck's disease were screened. Wrist plain X-ray and magnetic resonance imaging (MRI) were made for classification of patients according to the Lichtman criteria. After exclusion, we were left with 24 subjects who were included in the study. Demographic data included age and sex.

**Figure 1:**
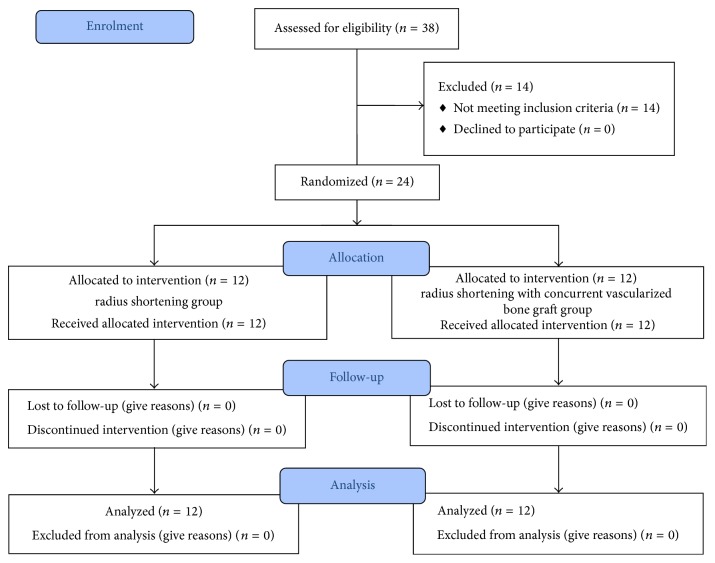
Study design flowchart.

Eligible subjects were assigned to radial shortening group (A) or radial shortening combined with vascularized bone graft group (B) (Figure 1).

### 2.2. Procedures

All the operations were carried out under general anesthesia. The patients were in the supine position and the forearm was in supination with the extremity put over a side table. We used a volar longitudinal incision match to the distal part of Henry's approach [19]. After cutting the superficial fascia, the wrist is flexed, the radial artery is drawn back laterally, and the flexor carpi radialis and flexor pollicis longus are drawn back medially. All of the patients had ulnar minus variance before the surgery.

#### 2.2.1. Group A, Shortening of the Radius

**Figure 2:**
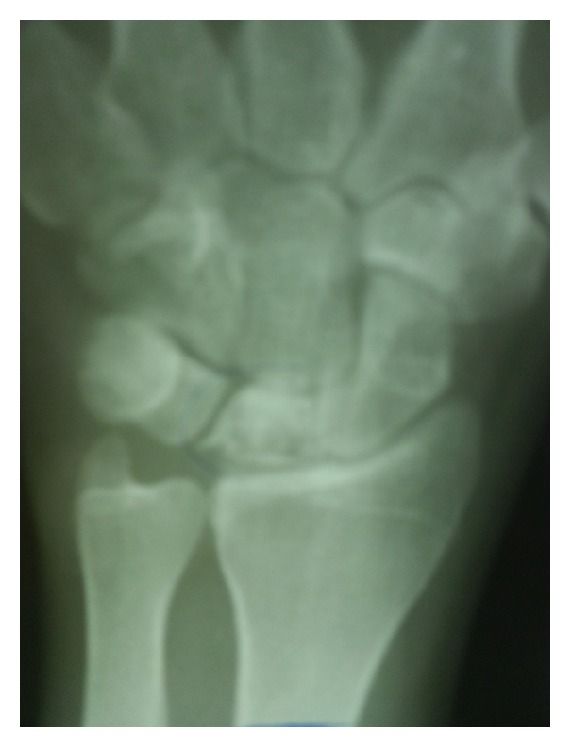
Preoperative radiogram of a 33-year-old male with Lichtman type IIIA Kienböck's disease.

**Figure 3:**
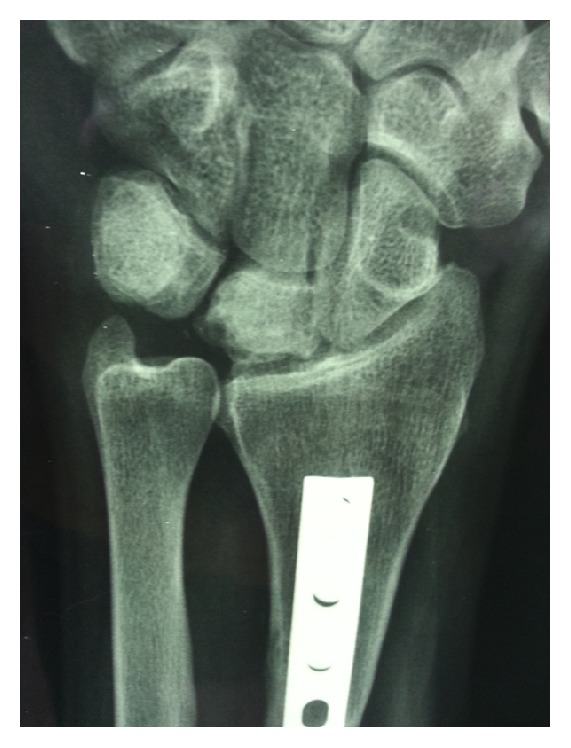
Postoperative radiogram of a 33-year-old male with Lichtman type IIIA Kienböck's disease.

The pronator quadratus muscle is raised from lateral border of the radius and lie in a horizontal position carefully. The shortening osteotomy was calculated on standard radiographs in neutral position before the surgery and was no longer than 3 mm. The level of the osteotomy was at the diaphyseal area, nearly 7 cm proximal to the distal articular surface of the radius bone. The osteotomy is fixed with a 3.5 mm dynamic compression plate. At the end, the pronator quadratus muscle is sutured back over the plate (Figures 2 and 3).

#### 2.2.2. Group B, Shortening of the Radius Combined with Vascularized Bone Graft

For dissection of the graft, we began with an incision in the fascia and the distal part of the pronator quadratus which allows a suitable exposure of the distal aspect of the radius. The incision in periosteum was along the proximal and distal margins of a 1 cm strip of muscle and fascia. The radial half of the strip was raised with its periosteum off the radius volar cortex. The dissection was done subperiosteally up to the radial artery on the lateral aspect of the pedicle. The harvesting of graft was done by using 5 mm osteotomes. The graft was based on carpal branch of radial artery between radial and ulnar arteries, and its pedicle was dissected to the transverse volar carpal artery origin. After that the radial shortening was done as described for group A, and then placement of vascularized bone graft was done. For placement of vascularized bone graft, the wrist joint capsule was incised and the bone graft was then placed in an about 4 mm deep and wide hole which was made in the middle of the lunate bone. The stabilization was done with a fine k-wire and the capsule was closed in a loose way in order to minimize the compression on the pedicle.

Single dose first generation cephalosporin was used for prophylaxis preoperatively. Postoperative immobilization was applied with a short arm splint for four weeks. After four weeks, the k-wire was removed and the wrist motion started gradually. Wrist plain X-ray was taken at weeks 6 and 12.

### 2.3. Variables Assessment

For assessment of outcome of surgery in two groups, we used the Mayo Wrist score. It is one of the most commonly used wrist scores with international application. It is answered on a scale from 0 to 100 and consists of four parts, including pain intensity (0–25), function (0–25), range of motion (0–25), and grip strength (0–25) [20]. The Mayo Score was used before and nine months after the surgery.

### 2.4. Blinding

Randomization was done by a third party physician using tables of random numbers. Mayo Wrist score was assessed by a physician who was not informed about method of surgery of the patients.

### 2.5. Statistical Analysis

The data were analyzed by Fisher's exact test and independent *t*-test for demographic differences between two groups. For comparing the Mayo Wrist score between and within two groups, before and after surgery, we used repeated measure of ANOVA and paired sample *t*-test. Comparison of the subjects' frequency in different parts of Mayo Wrist score, before and after surgery, was carried out by Mann-Whitney *U* test and Wilcoxon signed-ranks test. The data were analyzed using Statistical Package for the Social Sciences version 20.0 (SPSS Inc., Chicago, Illinois, USA) and a *P* < 0.05 was considered statistically significant.

## 3. Results

### 3.1. Baseline Profile

Twenty-four patients completed the procedure. The mean (SD) age was 35.2 (4.9) years, ranging from 28 to 44 years old. The demographic features of the sample are reported in Table 1. Comparison of baseline profile of subjects, including age and sex, revealed no statistically significant differences between two groups.

Before surgery the mean Mayo Wrist score (SD) was 27.1 (15.4) in group A and 32.5 (18.3) in group B. The difference was not significant (*P* = 0.441).

Comparison of frequency of subjects in different scores of different parts of Mayo Wrist score showed that before the surgery there were not any significant differences between two groups.

### 3.2. Outcomes

After surgery the mean Mayo Wrist score (SD) was 74.6 (5.4) and 85.8 (5.1) for group A and group B, respectively. Paired sample *t*-test showed that the mean score increased in both groups significantly.

ANOVA analysis showed that, after surgery, the mean Mayo Wrist score was more in group B than in group A significantly (*P* = 0.001) (Table 2).

Comparison of frequency of subjects in different scores of different parts of Mayo Wrist score, before and after the surgery, is shown in Table 3. After the surgery the pain intensity, function status, range of motion, and grip strength were improved in both groups significantly.

The improvement in function status, range of motion, and grip strength in group B was significantly more than in group A, but the difference was not significant for pain intensity.

## 4. Discussion

For treatment of Kienböck's disease, there are various procedures available, either independently or in combinations. Radial shortening has been well accepted as effective surgical procedure for Kienböck's disease. Also, vascularized bone grafting is an attractive method which improves the local biological environment and thereby may promote the revascularization. In this study, we compared radial shortening method with radial shortening combined with vascularized bone graft in patients with stage II or IIIA of Kienböck's disease.

The results showed that radial shortening is efficient in reducing pain and in improving the function, range of wrist motion, and grip strength. This is consistent with most of previous studies which presented this method as a reliable procedure for functional improvement and patient satisfaction in the treatment of stages I to IIIA of Kienböck's disease [7, 11, 14, 21, 22].

We also concluded that radial shortening in combination with vascularized bone graft can reduce the pain and improve the function, range of wrist motion, and grip strength effectively in patients. This is the same as in Zafra et al., Waitayawinyu et al., and Mathoulin et al. conclusions which used combination of shortening osteotomy and vascularized bone grafting for the treatment of Kienböck's disease [16, 17, 19].

Our study showed that, in comparison with radial shortening alone, when radial shortening is combined with vascularized bone graft, there is even more improvement in Mayo Wrist score and final outcome of surgery in factors of function, range of motion, and grip strength. The reducing of pain was not significantly different between two groups.

In two open label trails,, Zafra et al. and Mathoulin et al. concluded that there are good clinical results with the combination of shortening of the radius combined with vascularized bone graft [17, 19]. In another study, Arora et al. used vascularized iliac bone graft for reconstruction of Kienböck's disease and showed that this is a reasonable method and radiological and clinical improvements last for a long period of time [23].

On the other hand, Afshar and Eivaziatashbeik compared long-term outcomes of radial shortening osteotomy versus pedicled vascularized bone graft and concluded that there was no considerable clinical or radiological difference between these two surgical methods in long-term outcome [15].

In Zafra et al. and Mathoulin et al. studies, radial shortening and vascularized bone graft were assessed as a combined method but not in comparison with other methods; so there is a bias that an osteotomy alone or the graft alone could have perhaps given the same results. And in Afshar and Eivaziatashbeik study radial shortening alone and vascularized bone graft alone were compared together [15, 17, 19].

But in this study, we compared radial shortening with a combination of radial shortening and vascularized bone graft. So in contrast with Afshar and Eivaziatashbeik study which stated that vascularized bone graft alone has no priority to radial shortening, we concluded that combination of shortening and graft is more efficient than shortening alone. This may be because of augmentation of treatment when using these two modalities concurrently, instead of using each one alone.

In conclusion, the authors believe that radial shortening combined with vascularized bone graft is a valuable method that presents clinical improvements in the treatment of early stages of Kienböck's disease. This method can be more effective than traditional methods like radial shortening, in functional and clinical improvement of patients after surgery.

In this study, the follow-up was limited to assessment of clinical outcomes and final outcomes of reconstruction of lunate bone are not completely obvious. More works with longer time of follow-up and radiologic assessments are needed to conclude whether this surgical option represents a long-time improvement over conventional treatment alternatives.

## Figures and Tables

**Table 1:** Demographics and clinical characteristics of subjects (*n* = 24).

Characteristics	Radial shortening	Radial shortening and vascularized bone graft	*P*
	*N* = 12	*N* = 12	
Age, mean (SD) year	35.7 (5.2)	34.6 (4.8)	0.604
Sex			
Male	10 (83.3)	10 (83.3)	1.000
Female	2 (16.7)	2 (16.7)	

All variables are Number (%) unless otherwise indicated.

*P*: *P*-value is extracted from Independent Samples Test and Fisher's Exact Test.

**Table 2:** Comparison of mean Mayo Wrist score, between and within groups, before and after the surgery (*n* = 24).

Characteristic	Radial shortening		Radial shortening and graft		Between groups	
	*N* = 12		*N* = 12		*P*-value	
	Before surgery	After surgery	Before surgery	After surgery	Before surgery	After surgery
Mean Mayo Wrist score (SD)	27.1 (15.4)	74.6 (5.4)	32.5 (18.3)	85.8 (5.1)	0.441	0.001
Within groups *P*-value	<0.001		<0.001			

*P*: *P*-value is extracted from Repeated Measure of ANOVA and Paired sample *t*-test.

**Table 3:** Comparison of frequency of subjects in different scores of different parts of Mayo Wrist score, before and after the surgery (*n* = 24).

Mayo Wrist score parts	Radial shortening		Radial shortening and graft		Between groups	
	*N* = 12		*N* = 12		*P*-value	
	Before surgery	After surgery	Before surgery	After surgery	Before surgery	After surgery
Pain intensity						
No pain	0	0	0	0	1.000	0.75
Mild occasional	0	11 (91.7)	0	10 (83.3)		
Moderate, tolerable	6 (50)	1 (8.3)	6 (50)	2 (16.7)		
Sever to intolerable	6 (50)	0	6 (50)	0		
**Within groups P-value**	0.002		0.001			
Function status						
Returned to regular employment	0	0	0	6 (50)	0.32	0.03
Restricted employment	3 (25)	12 (100)	6 (50)	6 (50)		
Able to work, but unemployed	0	0	0	0		
Unable to work because of pain	9 (75)	0	6 (50)	0		
**Within groups P-value**	0.003		0.007			
Range of motion (% of normal side)						
100%	0	0	0	0	0.75	0.001
75–99%	0	3 (25)	0	11 (91.7)		
50–74%	0	9 (75)	0	1 (8.3)		
25–49%	11 (91.7)	0	12 (100)	0		
0–24%	1 (8.3)	0	0	0		
**Within groups P-value**	0.001		0.001			
Range of motion (If only injured hand examined)						
Greater than 120 degrees	0	0	0	0	1.000	0.03
90–120 degrees	0	6 (50)	0	11 (91.7)		
60–90 degrees	0	6 (50)	0	1 (8.3)		
30–60 degrees	12 (100)	0	12 (100)	0		
less than 30 degrees	0	0	0	0		
**Within groups P-value**	0.002		0.001			
Grip strength % of normal						
100%	0	0	0	0	1.000	0.001
75–99%	0	3 (25)	0	12 (100)		
50–74%	0	9 (75)	0	0		
25–49%	12 (100)	0	12 (100)	0		
0–24%	0	0	0	0		
**Within groups P-value**	0.001		0.001			

All variables are Number (%).

*P*: *P*-value is extracted from Mann-Whitney Test and Wilcoxon Signed Ranks Test.
